# LTR point mutations in the *Tax*-responsive elements of HTLV-1 isolates from HIV/HTLV-1-coinfected patients

**DOI:** 10.1186/1743-422X-9-184

**Published:** 2012-09-04

**Authors:** Mariana Cavalheiro Magri, Emanuela Avelar Silva Costa, Adele Caterino-de-Araujo

**Affiliations:** 1 Immunology Department, Instituto Adolfo Lutz, Av. Dr. Arnaldo, 355, 11° andar, São Paulo, SP, 01246-902, Brazil; 2 Faculdade de Ciências Farmacêuticas, Universidade de São Paulo, Av. Prof. Lineu Prestes 580, São Paulo, SP, CEP 05508-000, Brazil

**Keywords:** HTLV-1, LTR region, Sequencing

## Abstract

**Background:**

In *Virology Journal* 2011, 8:535, Neto et al. described point mutations into *Tax*-responsive elements (TRE) of the LTR region of HTLV-1 isolates from asymptomatic carriers from Sao Paulo, Brazil, and hypothesized that the presence of the G232A mutation in the TRE-1 increase viral proliferation and consequently the proviral load (PvL), while the A184G mutation in the TRE-2 do not have such effect.

**Findings:**

We performed the real-time PCR assay (*pol*) and sequenced LTR region of HTLV-1 isolates from 24 HIV/HTLV-1-coinfected patients without HTLV-1-associated diseases from the same geographic area. These sequences were classified as belonging to the transcontinental subgroup A of the Cosmopolitan subtype a. The frequency of G232A mutation (16/24, 66.7%) was high as much as 61.8% reported by Neto’s in HTLV-1 asymptomatic carriers with high PvL. High frequency (13/24, 54.2%) of double mutations G232A and A184G was also detected in HIV/HTLV-1-coinfected patients. We did not quantify PvL, but comparative analyses of the cycle threshold (Ct) median values of the group of isolates presenting the mutated-types sequences (Ct 33.5, n = 16) versus the group of isolates with the wild-type sequences (Ct 32, n = 8) showed no statistical difference (*p* = 0.4220).

**Conclusion:**

The frequencies of mutated-type sequences in the TRE-1 and TRE-2 motifs were high in HIV/HTLV-1-coinfected patients from Sao Paulo, Brazil. If these LTR point mutations have predictive value for the development of HTLV-1-associated diseases or they correspond to the subtype of virus that circulate in this geographic area has to be determined.

## Findings

In the December 13^th^, 2011 issue of *Virology Journal*, Neto and collaborators described point mutations into 21-bp repeat motifs, named *Tax*-responsive elements (TRE), present in the 5’ LTR region of human T-lymphotropic virus type 1 (HTLV-1) isolated among asymptomatic carriers from Sao Paulo, Brazil, and hypothesized that the presence of G232A nucleotide substitution in the A domain of the first TRE (TRE-1) increase viral proliferation and consequently the proviral load (PvL), while the A184G mutation in the A domain of TRE-2 do not have such effect [1]. This hypothesis is very interesting, and we decided to add some information in regard to the presence of such point mutations in HIV/HTLV-1-coinfected patients who present no symptoms of HTLV-1-associated diseases from the same geographic area.

A previous study conducted by our group working at the Instituto Adolfo Lutz (a public health laboratory in Sao Paulo, Brazil) analyzed the best algorithm to be employed in the diagnosis of HTLV-1 and HTLV-2 in at-risk individuals of Sao Paulo. We performed confirmatory assay by the real-time PCR (at least in duplicate) to detect *pol* provirus gene segments of HTLV-1 and HTLV-2, and employed the human albumin gene as an internal control. The real-time PCR (*pol*) optimization for HTLV-1 was conducted with the cell line C91-PL using 10-fold DNA dilutions, and showed detection limit of 10 ng of DNA input. We did not quantify the proviral load (PvL), and used the PCR as qualitative assay. The coefficient of variation of the cycle threshold (Ct) values for the intra- and inter-assays were less than 1.6% and 1.0%, respectively [2].

At present study, DNA samples of 24 HIV/HTLV-1-coinfected individuals of which we have Ethnical Board approval and that scored HTLV-1 positive by the real-time PCR assay (*pol*) were also sequenced for partial LTR region, and by NCBI-Genotyping and REGA-Subtyping tools websites and phylogeny they were classified as belonging to the transcontinental subgroup A of the Cosmopolitan subtype a, the same subgroup A detected by us in a previous study conducted with HIV/HTLV-1-coinfected patients [3], and by Neto et al. among asymptomatic carriers [1]. The median age of the 24 individuals was 44 years (ranging from 27 to 65) and 58.3% were female (Table 1). Multiple alignment of the 24 sequences [GenBank:JF271843 to JF271852, and JX280947 to JX280960] along with the ATK prototype (wild-type sequence) [GenBank:J02029] and the TRE-1 and TRE-2 motifs described by Neto et al. were performed using the Clustal W multiple-sequence alignment tool from BioEdit Sequence Alignment Editor version 7.0.5.3 software.

**Table 1:** General features of HIV-HTLV-1-coinfected individuals from Sao Paulo, Brazil

**Sample(n = 24)**	**GenBank(AN)**	**Gender**	**Age(years)**	**Real-time PCR**		**Sequencing (LTR)**	
				**HTLV-1 (*pol*)**	**Albumin**	**TRE-1 motif**	**TRE-2 motif**
				**Ct**	**Ct**	**AAGGCTCTGACGTCTCCCCCC**	**TAGGCCCTGACGTGTCCCCC**
BRSP134-08	JF271843	M	45	37	35	**.**	**.**
BRSP145-08	JF271844	M	50	36	30	G	A
BRSP206-08	JF271845	F	42	34	29	G	**.**
BRSP232-08	JF271846	F	56	35	27	G	A
BRSP42-09	JF271847	F	55	33	28	**.**	**.**
BRSP205-09	JF271848	F	27	36	27	G	A
BRSP320-09	JF271849	F	51	36	31	**.**	**.**
BRSP414-09	JF271850	M	62	40	25	G	A
BRSP425-09	JF271851	F	57	32	26	**.**	**.**
BRSP01-10	JF271852	F	32	41	32	G	A
BRSP30-10	JX280947	F	38	29	24	**.**	**.**
BRSP37-10	JX280948	M	43	29	24	G	A
BRSP38-10	JX280949	M	45	32	25	G	**.**
BRSP71-10	JX280950	M	33	28	21	G	A
BRSP93-10	JX280951	F	43	27	22	G	A
BRSP97-10	JX280952	M	37	33	22	G	A
BRSP101-10	JX280953	F	40	30	24	**.**	**.**
BRSP105-10	JX280954	M	41	29	22	G	A
BRSP118-10	JX280955	M	65	27	22	**.**	**.**
BRSP171-10	JX280956	F	30	32	22	**.**	**.**
BRSP40-11	JX280957	M	46	36	25	G	A
BRSP142-11	JX280958	F	57	32	24	G	**.**
BRSP194-11	JX280959	F	37	33	24	G	A
BRSP69-12	JX280960	F	50	36	30	G	A

Legend: *AN*-Accession Numbers, *F*-Female, *M*-Male, *Ct*- Cycle threshold.

The frequency of the substitution A → G in the second nucleotide (A domain) of the TRE-1 motif (the TRE-1 G232A mutation) and the substitution G → A in the third nucleotide (A domain) of the TRE-2 motif (the TRE-2 A184G mutation) was summarized in Table 1. Of 24 subjects, the TRE-1 G232A mutation was detected in 16 (66.7%) and the TRE-2 A184G mutation in 13 (54.2%). Furthermore, 13 subjects (54.2%) had double mutations of the TRE-1 G232A and the TRE-2 A184G, three (12.5%) had the TRE-1 G232A mutation only, none had the TRE-2 A184G mutation only, and eight (33.3%) had no mutation in both sites. Thus, all the 13 subjects with the TRE-2 A184G mutation had the TRE-1 G232A mutation together.

The frequency of the TRE-1 G232A mutation (66.7%) in HIV/HTLV-1-coinfected patients was high as much as 61.8% reported by Neto’s in HTLV-1 asymptomatic carriers with a high PvL [1]. We also found that the frequency of double mutations of the TRE-1 G232A and the TRE-2 A184G in HIV/HTLV-1-coinfected was high (54.2%). Unfortunately we did not quantify PvL, thus we could not compare PvL between patients with and without TRE point mutations. Instead, we compared the Ct median value of the real-time PCR assay (*pol*) between the two groups. There was no statistical difference in the median of the Ct values between patients with the any mutated-types sequences of 33.5 (ranging from 27 to 41, n = 16) and patients with the wild-type sequences of 32 (ranging from 27 to 37, n = 8) (Mann–Whitney test, *p* = 0.4220).

Regardless the limited number of samples of our study we confirmed the frequency of mutated-type sequences in the TRE-1 and TRE-2 was high in HIV-HTLV-1-coinfected patients. We do not know if these mutated-type sequences are the result of a selective advantage of such virus during chronic infection or if they denote the types of HTLV-1 that circulate in this geographic area. Interestingly, in our ongoing study, when we compare LTR sequences from Brazil and sequences from elsewhere, mutated-type sequences in the TRE-1 and TRE-2 regions are detected mostly in Brazil (data not shown). In opposition of what is hypothesized by Neto et al., we suggest that these LTR point mutations could have a protective effect, since no case of HTLV-1-associated diseases was detected among such patients, although a longitudinal study is necessary to confirm this hypothesis. Yet, it is relevant to point that all the individuals from the present study were undergoing antiretroviral therapy, which could interfere in retrovirus selection. Corroborating our hypothesis, Montagne et al. studying the effect of point mutations in the A, B and C domains of TRE-3 observed that the integrity of the B domain was absolutely required to mediate the Tax1 activation of the 21-bp enhancer element, while mutation of the A and C domains reduced the Tax1 effect, and mutation in both A and C domains abolished the Tax1 effect [4]. Thus, the integrity of the TRE is imperative for the induction of the promoter / enhancer element, and consequently disease progression.

Taking together these data we do not know if the high PvL precede the emergence of the mutated-type sequences or is a consequence of the presence of such mutated strains. Only longitudinal studies could help to answer this question. Finally, it is important to extend these data to clarify the role of the LTR point mutations in TRE, and to identify markers that could have predictive value for the development of HTLV-1-associated diseases, mostly in countries were HTLV-1 is endemic, like Brazil.

## Consent

The Ethical Board of Instituto Adolfo Lutz approved this work. Written informed consent to publish the data in the Table was obtained from all the patients.

## Abbreviations

TRE: *Tax*-responsive element; LTR: Long-terminal repeat; HTLV-1: Human T-cell lymphotropic virus type 1; PvL: Proviral load; PCR: Polymerase chain reaction; Ct: Cycle threshold.

## Competing interest

The authors declare that they have no competing interests.

## Authors’ contributions

MCM conceived the study, conducted the characterization of the LTR sequences and data analysis of the sequences, and wrote the manuscript. EASC did the real-time PCR assays. ACA was responsible of scientific version, discussion and interpretation of data. All authors read and approved the final manuscript.

## Authors’ information

MCM present address: Laboratório de Hepatologia por Vírus, Hospital das Clínicas, Faculdade de Medicina, Universidade de São Paulo, S.P., Brasil.

## References

[B1] Neto WK Da-Costa AC Oliveira ACS Martinez VP Nukui Y Sabino E Sanabani SS Correlation between LTR point mutations and proviral load levels among Human T cell Lymphotropic Virus type 1 (HTLV-1) asymptomatic carriers Virol J 2011 8 535 10.1186/1743-422X-8-535 22166003 PMC3287369

[B2] Costa EAS Magri MC Caterino-de-Araujo A The best algorithm to confirm the diagnosis of HTLV-1 and HTLV-2 in at-risk individuals from São Paulo, Brazil J Virol Methods 2011 173 280 286 10.1016/j.jviromet.2011.02.018 21349293

[B3] Magri MC Brigido LFM Rodrigues R Morimoto HK Ferreira JLP Caterino-de-Araujo A Phylogenetic and Similarity Analysis of HTLV-1 Isolates from HIV-Coinfected Patients from the South and Southeast Regions of Brazil AIDS Res Hum Retroviruses 2012 28 1 110 114 10.1089/aid.2011.0117 21591992 PMC3252815

[B4] Montagne J Beraud C Crenon I Lombard-Platet G Gazzolo L Sergeant A Jalinot P Tax1 induction of the HTLV-l 21 bp enhancer requires cooperation between two cellular DNA-binding protein EMBO J 1990 9 3 957 964 2311587 10.1002/j.1460-2075.1990.tb08194.x PMC551758

